# Bayesian Methods for Information Borrowing in Basket Trials: An Overview

**DOI:** 10.3390/cancers16020251

**Published:** 2024-01-05

**Authors:** Tianjian Zhou, Yuan Ji

**Affiliations:** 1 Department of Statistics, Colorado State University, Fort Collins, CO 80523, USA; 2 Department of Public Health Sciences, University of Chicago, Chicago, IL 60637, USA

**Keywords:** basket trial, Bayesian method, borrow information, drug development, hierarchical model, oncology, shrinkage estimation, prior

## Abstract

**Simple Summary:**

This paper provides a review of statistical methods for tumor-agnostic clinical trials. In particular, the review focuses on basket trials and provides methodological insights into various Bayesian approaches. The key concept of borrowing information through Bayesian hierarchical models is emphasized, and some novel trial designs are introduced. The review is expected to provide oncology and biostatistics researchers with more exposure to powerful Bayesian methods for the design and analysis of tumor-agnostic clinical trials.

**Abstract:**

Basket trials allow simultaneous evaluation of a single therapy across multiple cancer types or subtypes of the same cancer. Since the same treatment is tested across all baskets, it may be desirable to borrow information across them to improve the statistical precision and power in estimating and detecting the treatment effects in different baskets. We review recent developments in Bayesian methods for the design and analysis of basket trials, focusing on the mechanism of information borrowing. We explain the common components of these methods, such as a prior model for the treatment effects that embodies an assumption of exchangeability. We also discuss the distinct features of these methods that lead to different degrees of borrowing. Through simulation studies, we demonstrate the impact of information borrowing on the operating characteristics of these methods and discuss its broader implications for drug development. Examples of basket trials are presented in both phase I and phase II settings.

## 1. Introduction

The field of tissue-agnostic drug development has seen increasing interest due to recent advances in molecular genetics and biomarker-driven treatment strategies. Basket trials, a type of clinical trial, have gained particular attention in this area since they simultaneously evaluate a single therapy across multiple cancer types or subtypes of the same cancer [1,2,3,4,5,6]. The rationale behind basket trials is that treatments targeting specific molecular alterations can potentially treat tumors regardless of their origin in the body. By using master protocols, basket trials can enhance operational efficiency and increase patient participation. Examples of basket trials include a study of imatinib in multiple histological subtypes of advanced sarcoma [7], a study of vemurafenib in BRAF V600 mutation-positive non-melanoma cancers [8], and a study of larotrectinib in TRK fusion-positive cancers [9], among others.

The mechanism of a drug in a basket trial is based on modifying a cancer biomarker that is prevalent across different cancer types or subtypes. Instead of conducting one trial for one disease, a basket trial includes multiple baskets, each representing a disease, so that the drug efficacy can be tested at the same time across baskets in a single clinical trial. In a way, a basket trial can be seen as a collection of multiple single-arm subtrials, one for each disease. There are typically no control arms in a basket trial, and patients are enrolled in parallel. An obvious benefit of basket trials is that only one study team and study protocol are needed for a trial. In a basket trial, the statistical analysis of each substudy can be independent of the others. This is known as stratified analysis. However, if a basket has a small sample size, e.g., for a rare cancer type, the stratified analysis often results in large uncertainty and lacks sufficient power for efficacy evaluation. To mitigate this issue, it may be desirable to borrow information across baskets since the same treatment is tested across all of them. This enables the treatment effect in one basket to be informed by the treatment effects in other baskets, leading to improved statistical precision and power. The Bayesian paradigm provides a natural way to achieve information borrowing. For example, by assuming a common prior distribution on the basket-specific response rates, their estimates are shrunk toward a common value and tend to have lower variances.

Numerous Bayesian methods [10,11,12,13,14,15,16,17,18,19,20,21,22,23] have been developed to facilitate information sharing for basket trials, or more broadly, in clinical trials involving multiple patient subpopulations. We review the common components of these methods, such as a sampling model for the numbers of responders which involves the response rates as parameters, a transformation applied to the response rates, a prior model for the transformed response rates which typically expresses an assumption of exchangeability, a criterion for selecting the promising baskets, and a possible interim analysis plan. With the same general components, these methods mainly differ by the transformation applied to the response rates, which reflects *what* information is borrowed, and the prior model for the transformed response rates, which determines *how* information is borrowed. For example, some methods directly borrow the raw response rates, while others borrow the response rate increments from the reference rates; some methods model the transformed response rates as a random sample from a unimodal distribution, while others utilize multimodal mixture distributions. We discuss the impact of these modeling choices, particularly those related to the degree of borrowing, on the operating characteristics of the methods.

The methods for information borrowing can be extended and applied to trials involving both multiple diseases and multiple doses. Such extensions are useful for dose optimization trials under the recent Project Optimus initiative launched by the U.S. Food and Drug Administration (FDA) [24,25]. For example, after a dose escalation stage, multiple doses may be considered for expansion in multiple disease indications [26,27]. In this case, the baskets are nested in the dose expansion cohorts. We review some recent developments in this area [28].

The remainder of this paper is structured as follows. In Section 2, we review the general components, possible modeling choices, and operating characteristics of Bayesian methods for basket trials in a phase II setting. In Section 3, we review some recent developments of Bayesian methods for basket trials in the context of phase I dose optimization, which accommodate both multiple diseases and multiple doses. In Section 4, we present a discussion on future directions. Finally, Section 5 encapsulates our conclusions.

## 2. Basket in Phase II Studies

### 2.1. Trial Examples

A large number of basket trials are conducted in an exploratory phase II setting. The primary endpoint is typically tumor response. If the drug is deemed promising in some cancer (sub)types, it would warrant further investigation in a confirmatory study or conditional marketing approval.

We review three such trials. The first trial was aimed at assessing the efficacy of imatinib in patients with one of 10 different subtypes of advanced sarcoma [7]. These included angiosarcoma, Ewing sarcoma, fibrosarcoma, leiomyosarcoma, liposarcoma, malignant fibrous histiocytoma (MFH), osteosarcoma, malignant peripheral-nerve sheath tumor (MPNST), rhabdomyosarcoma, and synovial sarcoma. The primary endpoint was tumor response, defined as complete response (CR) or partial response (PR) at 2 months, or stable disease, CR or PR at 4 months. The trial was designed based on a Bayesian hierarchical model [10]. Table 1 summarizes the patient responses by sarcoma subtype. A total of 179 patients were available for analysis. By comparing the response rates to a reference rate of 30%, the authors concluded that imatinib was not an active agent in advanced sarcoma in these subtypes.

The second trial was conducted to study vemurafenib in BRAF V600 mutation-positive non-melanoma cancers. The study included the following cancer cohorts that received vemurafenib monotherapy: non-small-cell lung cancer (NSCLC), cholangiocarcinoma (CCA), Erdheim–Chester disease or Langerhans’ cell histiocytosis (ECD/LCH), anaplastic thyroid cancer, breast cancer, ovarian cancer, multiple myeloma, colorectal cancer (CRC-V), and all others. An additional cohort of patients with colorectal cancer received vemurafenib combined with cetuximab (CRC-VC). The primary endpoint was tumor response at week 8, as assessed by the site investigators according to the Response Evaluation Criteria in Solid Tumors [29], or the criteria of the International Myeloma Working Group [30]. The trial was designed using Simon’s two-stage method [31,32], separately for each cohort. Due to insufficient accrual, patients in the breast cancer, multiple myeloma, and ovarian cancer cohorts were eventually included in the all-others cohort. Table 2 summarizes the patient responses by cohort (not including the all-others cohort). A total of 84 patients were available for analysis. By comparing the response rates to a reference rate of 15%, the authors concluded that BRAF V600 appeared to be a targetable oncogene in some, but not all, non-melanoma cancers. Specifically, preliminary vemurafenib activity was observed in NSCLC and in ECD/LCH.

The data from the imatinib and vemurafenib trials have since then been reanalyzed multiple times [12,16,21].

A third example is the recent pivotal study of larotrectinib [9]. A family of genes called NTRK1, NTRK2, and NTRK3 encode a protein called tropomyosin receptor kinases (TRK). Mutation in NTRK genes results in TRK fusion proteins that lead to tissue-independent oncogenic transformation [33,34,35]. TRK fusion proteins are found in more than 20 different tumor types. As a result, a phase II basket trial was conducted to evaluate the therapeutic effect of larotrectinib, a TRK inhibitor, in 55 patients diagnosed with 12 different cancer types. The overall response rate was 75% based on central assessment with a 95% confidence interval of (61%, 85%). Larotrectinib was well tolerated in both adult and child populations. Based on the efficacy and safety data, the drug has been approved for treating NTRK gene fusion-positive tumors in adult and pediatric patients across cancer types. Statistical analysis pooled all the patients enrolled in the trial regardless of their tumor types. Therefore, the baskets were not differentiated in the statistical inference of drug effects. This is a special case where the biomarker, NTRK gene fusion, is highly specific and causal to a small fraction of cancers, regardless of their tissue types. In general, a targeted therapy may work in some cancer types or subtypes, which requires more sophisticated statistical design and analysis.

### 2.2. Statistical Setup

Consider a basket trial with *J* baskets. Let $n_{j}$ denote the number of patients enrolled in basket *j*. The number of responders in basket *j*, denoted by $y_{j}$, is typically modeled by a binomial distribution,

(1) $$\begin{array}{c} y_{j}|n_{j},\pi_{j}∼\mathrm{Bin}\left(n_{j},\pi_{j}\right),\;j=1,\ldots ,J. \end{array}$$

Here, $\pi_{j}$ represents the true but unknown response rate of the treatment in basket *j*. The efficacy of the treatment can be evaluated by comparing $\pi_{j}$ to a prespecified reference rate $\pi_{0j}$ via a hypothesis test,

(2) $$\begin{array}{c} H_{0j}:\pi_{j}\leq \pi_{0j}\;\mathrm{vesus}\;H_{1j}:\pi_{j}>\pi_{0j}. \end{array}$$

This reference rate can vary across baskets due to different cancer (sub)types being considered. If the observed data show strong evidence in favor of $H_{1j}$, the null hypothesis $H_{0j}$ is rejected and the treatment is determined to be efficacious in basket *j*. Under the Bayesian paradigm, one assigns a prior distribution to $\pi_{j}$ and calculates its posterior distribution according to Bayes’ rule. The treatment is deemed promising in basket *j* if the posterior probability of the alternative hypothesis exceeds a prespecified threshold $q_{j}$ (e.g., 0.95),

$$\begin{array}{c} \mathrm{Pr}\left(\pi_{j}>\pi_{0j}|\mathrm{data}\right)>q_{j}. \end{array}$$

As mentioned earlier, it is desirable to specify a prior for $\pi_{j}$s which allows information borrowing across baskets. In the following sections, we discuss several considerations involved in the prior specification.

### 2.3. Prior Specification and Exchangeability

**Figure 1:**
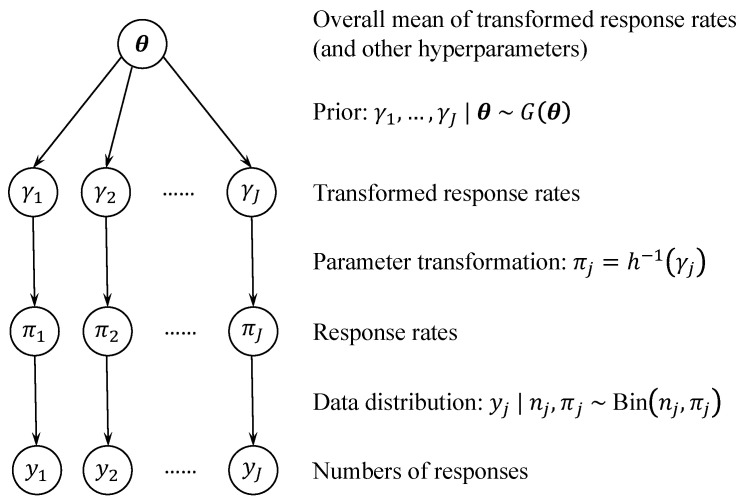
Graphical representation of the hierarchical model that allows information borrowing across baskets.

**Figure 2:**
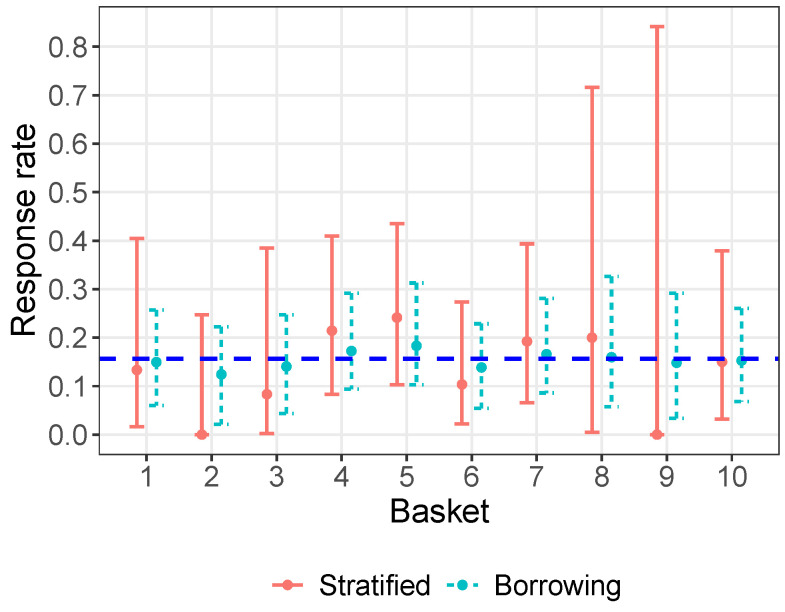
Illustration of the effect of information borrowing through an analysis of the imatinib data. Dots represent point estimates and error bars represent 95% confidence or credible intervals. In the stratified analysis, $y_{j}/n_{j}$ is used as a point estimate for $\pi_{j}$, and the Clopper–Pearson exact method is used to construct a confidence interval. For information borrowing, the following hierarchical model is used: $\gamma_{j}=\mathrm{logit}\left(\pi_{j}\right)-\mathrm{logit}\left(\pi_{0j}\right)$, $\gamma_{j}|\mu ,\sigma^{2}\overset{iid}{∼}N\left(\mu ,\sigma^{2}\right)$, $\mu ∼N(0,100^{2})$, σ∼Half-N(3). Then, the posterior mean of $\pi_{j}$ is used as its point estimate, and the 2.5th and 97.5th percentiles of its posterior distribution are used to form a credible interval. The dashed horizontal line corresponds to the observed overall response rate, 15.6%.

Most existing methods start by transforming $\pi_{j}$ into a real value using, for example, a logit transformation. We denote the transformation and the real-valued parameter as $\gamma_{j}=h\left(\pi_{j}\right).$ Note that each basket *j* is indexed by a different parameter $\gamma_{j}$. Then, the $\gamma_{j}$s are modeled as a random sample from a common population distribution *G*,

(3) $$\begin{array}{c} \gamma_{j}|\theta \overset{iid}{∼}G\left(\theta \right), \end{array}$$

where θ denotes the vector of hyperparameters that parameterize *G*. Figure 1 displays a graphical representation of the hierarchical model given by Equations (1) and (3). More discussions on the choices of *h* and G(θ) are deferred to Section 2.3 and Section 2.4. Importantly, θ is unknown and is estimated based on data from all baskets. As a result, the posterior of $\gamma_{j}$ is informed by both the responses within basket *j*, through the likelihood (1), and those in other baskets, through the prior (3). Figure 2 illustrates the effect of information borrowing through an analysis of the imatinib data in Table 1. The point estimates of $\pi_{j}$ for individual baskets are shrunk towards the overall response rate. Additionally, the interval estimates of $\pi_{j}$ have shorter lengths under borrowing compared to those under stratified analysis. More details of the analysis can be found in the caption of Figure 2.

Suppose a prior p(θ) is placed on θ. Implicit in model (3) is the (marginal) prior dependence among the $\gamma_{j}$s. Note that

(4) $$\begin{array}{c} p\left(\gamma_{1},\ldots ,\gamma_{J}\right)=\int \left[\prod_{j=1}^{J}p\left(\gamma_{j}|\theta \right)\right]p\left(\theta \right)d\theta \neq \prod_{j=1}^{J}p\left(\gamma_{j}\right). \end{array}$$

In fact, it can be shown that the $\gamma_{j}$s are positively correlated a priori [36], which enables information borrowing across baskets. Furthermore, model (3) implies an assumption of prior exchangeability among the $\gamma_{j}$s. Mathematically, a sequence of random variables is called *exchangeable* if their joint distribution is invariant to permutations. From Equation (4), the joint density $p(\gamma_{1},\ldots ,\gamma_{J})$ is invariant to permutations of the indexes (1,…,J). The assumption of prior exchangeability is reasonable when no information is available before the trial to claim that the treatment is more likely to be efficacious in certain baskets than others [37]. We note that modeling the $\gamma_{j}$s as independent draws from a common distribution is a stronger assumption than finite exchangeability: the former implies the latter, but not vice versa.

If there is prior knowledge to distinguish some $\gamma_{j}$s from others, one may incorporate an expanded notion of exchangeability in the prior construction. For example, historical clinical trials may suggest that the baskets can be divided into several subgroups. Each subgroup consists of baskets with similar historical success rates. Then, one may specify a separate prior model for the $\gamma_{j}$s within each subgroup. While the parameters within the same subgroup are exchangeable, those across different subgroups are not. This is known as partial exchangeability. For another example, patient responses are often associated with basket-level and patient-level covariates. If these covariates are available, they may be used to construct a regression model with an underlying assumption of conditional exchangeability. For the rest of this paper, we will restrict our attention to the exchangeable model given by Equation (3), which is employed by most existing methods.

### 2.4. What Information to Borrow?

The transformation $\gamma_{j}=h\left(\pi_{j}\right)$ reflects what information is borrowed across baskets. A straightforward choice is to directly borrow the response rates by assuming $\pi_{j}|\theta \overset{iid}{∼}G\left(\theta \right)$, where G(θ) is a distribution on the unit interval, e.g., a beta distribution. In this case, $h\left(\pi_{j}\right)=\pi_{j}$ is the identity transformation, and the underlying assumption is that the treatment has similar response rates across baskets. A variation in this choice is to consider a logit transformation, $h\left(\pi_{j}\right)=\mathrm{logit}\left(\pi_{j}\right)=\log\left[\pi_{j}/\left(1-\pi_{j}\right)\right]$. This can simplify posterior computation by allowing G(θ) to be a distribution over the real line, e.g., a normal distribution.

An alternative choice of *h* incorporates an adjustment for the reference rate $\pi_{0j}$. Typically, the reference rate for each basket is determined based on how well the cancer (sub)type responds to the standard of care. If there are substantial differences in the reference rates across baskets, it may be implausible to assume that the $\pi_{j}$s are similar. This is because baskets with lower (or higher) reference rates are also more likely to respond poorly (or positively) to the new treatment. To account for the differential reference rates, it may be more appropriate to model the response rate increments from the reference rates. For example, Berry et al. [11] considered borrowing the increments of the logit response rates, $h\left(\pi_{j}\right)=\mathrm{logit}\left(\pi_{j}\right)-\mathrm{logit}\left(\pi_{0j}\right)$.

Lastly, a different strategy is to borrow information at the hypothesis level by letting $\gamma_{j}=h\left(\pi_{j}\right)=1\left(\pi_{j}>\pi_{0j}\right)$. See, e.g., Zhou and Ji [21]. Here, $\gamma_{j}=1$ (or 0) represents $H_{1j}$ is true (or false), indicating that the treatment is efficacious (or inefficacious) in basket *j*. The prior G(θ) for $\gamma_{j}$s can be a Bernoulli distribution. Borrowing across $\gamma_{j}$s reflects the assumption that if the treatment is promising, it is likely to be efficacious across multiple baskets simultaneously. This is a more general assumption than assuming the response rates are similar. For example, $\pi_{j}$ and $\pi_{j^{\prime }}$ may be quite different, but as long as they are larger than $\pi_{0j}$ and $\pi_{0j^{\prime }}$, respectively, the treatment is efficacious in both baskets *j* and $j^{\prime }$. An additional complexity of this approach is that *h* is a many-to-one transformation, and the value of $\pi_{j}$ cannot be uniquely determined by $\gamma_{j}$ through $\pi_{j}=h^{-1}\left(\gamma_{j}\right)$. Instead, one needs to construct a prior for $\pi_{j}$ conditional on the value of $\gamma_{j}$. For example, $\pi_{j}|\gamma_{j}=0$ can be a beta distribution truncated to the interval $\left[0,\pi_{0j}\right]$, and $\pi_{j}|\gamma_{j}=1$ can be a beta distribution truncated to the interval $\left(\pi_{0j},1\right]$. The prior $p(\pi_{j}|\gamma_{j})$ establishes the connection between $\gamma_{j}$ and $\pi_{j}$ in Figure 1.

### 2.5. How Is Information Borrowed?

The choice of the prior G(θ) determines how information is borrowed across baskets. To illustrate ideas, suppose $\gamma_{j}$ is real-valued, e.g., $\gamma_{j}=\mathrm{logit}\left(\pi_{j}\right)-\mathrm{logit}\left(\pi_{0j}\right)$. A natural choice of G(θ) is then a normal distribution,

(5) $$\begin{array}{c} \gamma_{j}|\mu ,\sigma^{2}\overset{iid}{∼}N\left(\mu ,\sigma^{2}\right), \end{array}$$

where the hyperparameter vector $\theta =(\mu ,\sigma^{2})$. The mean parameter μ represents a transformed version of the overall response rate of the treatment across all baskets. The basket-specific $\gamma_{j}$s are shrunk toward the common μ. The variance parameter $\sigma^{2}$ controls the degree of borrowing, with smaller values implying stronger shrinkage effects. At one extreme, when $\sigma^{2}=0$, all $\gamma_{j}$ values must be equal. At the other extreme, when $\sigma^{2}$ approaches infinity, the shrinkage effects become negligible. The estimation of $\sigma^{2}$ plays a crucial role in the statistical analysis. On the ond hand, overestimating $\sigma^{2}$ may lead to inadequate borrowing, diminishing the benefits of shrinkage estimation. On the other hand, underestimating $\sigma^{2}$ may result in excessive borrowing, leading to inflated type I error rates and potential failures in drug development (more on this point in Section 2.6). Yet, due to the typically limited number of baskets in a basket trial, accurate estimation of $\sigma^{2}$ is a challenging task.

Taking a full Bayesian approach, a hyperprior is assigned to $\sigma^{2}$. A computationally convenient choice is the inverse-gamma prior, $\sigma^{2}∼\mathrm{IG}\left(\alpha ,\beta \right)$. See, e.g., Thall et al. [10] and Berry et al. [11]. It is commonly thought that small values of α and β produce a noninformative prior for $\sigma^{2}$. However, Gelman [36] showed that even with small values of α and β, the IG(α,β) prior could still be quite informative and might lead to underestimation of $\sigma^{2}$. Instead, the author advocated the use of a half-*t* prior as a less informative choice for the hierarchical standard deviation parameter, σ∼Half-*t_ν_*(*A*), with small ν and large *A*. Here, ν is the number of degrees of freedom, and *A* is the scale parameter. Special cases of the half-*t* prior include the half-Cauchy (when ν=1) and half-normal (when ν=∞) priors. The half-*t* prior was used by Neuenschwander et al. [12] and Zhou and Ji [21].

Alternatively, Chu and Yuan [14] proposed an empirical Bayesian approach to specify the value of $\sigma^{2}$ based on a measure of homogeneity among the baskets. The relationship between $\sigma^{2}$ and the homogeneity measure is determined through a simulation-based calibration procedure.

To further reduce the risk of excessive borrowing, the normal distribution prior in Equation (5) may be replaced by a distribution with heavier tails, e.g., a *t*-distribution. Such a prior accommodates occasional extreme parameters. In a basket trial, the response rates in a few baskets may be quite different from the others. A heavy-tailed prior still shrinks these extreme response rates toward the overall mean but avoids pulling them too much [37].

#### 2.5.1. Mixture Models

In some basket trials, patient responses across baskets exhibit a clustering structure. For example, in the vemurafenib trial (Table 2), the ECD/LCH and NSCLC cohorts have similar proportions of responses, suggesting they can be clustered together. The same applies to the CRC-V and CRC-VC cohorts. To exploit such a clustering structure, a multimodal mixture prior can be placed on $\gamma_{j}$ [12,15,18,21]. For example, consider G(θ) to be a mixture of normal distributions,

(6) $$\begin{array}{c} \gamma_{j}|\mu ,\sigma^{2},w,K\overset{iid}{∼}\sum_{k=1}^{K}w_{k}\cdot N\left(\mu_{k},\sigma_{k}^{2}\right). \end{array}$$

In this case, the hyperparameter vector $\theta =(\mu ,\sigma^{2},w,K)$ with $\mu =(\mu_{1},\ldots ,\mu_{K})$, $\sigma^{2}=\left(\sigma_{1}^{2},\ldots ,\sigma_{K}^{2}\right)$, and $w=(w_{1},\ldots ,w_{K})$. Here, *K* is the number of mixture components, and $w_{k}$, $\mu_{k}$, and $\sigma_{k}^{2}$ are the weight, mean, and variance of mixture component *k*, respectively. The weights satisfy $\sum_{k=1}^{K}w_{k}=1$.

To facilitate interpretation, observe that the mixture prior in Equation (6) is equivalent to the following hierarchical prior,

(7) $$\begin{array}{c} \gamma_{j}|\mu ,\sigma^{2},K,s_{j}=k∼N\left(\mu_{k},\sigma_{k}^{2}\right),\;\mathrm{Pr}\left(s_{j}=k|w,K\right)=w_{k}. \end{array}$$

In other words, each basket can be thought of as belonging to one of *K* latent subgroups. The indicator $s_{j}\in \left\{1,\ldots ,K\right\}$ denotes the subgroup membership for basket *j*, and $w_{k}$ represents the prevalence of subgroup *k*. Conditional on the subgroup memberships, information borrowing only occurs within each subgroup. Therefore, compared to the simple normal prior, the normal mixture prior allows for more judicious information borrowing. Specifically, in the presence of substantial heterogeneity among baskets, the normal mixture prior usually leads to less borrowing, reducing the risk of type I error rate inflation. Note that the subgroup memberships are unknown a priori, and all baskets share the same prior probability of belonging to any given subgroup. As a result, prior exchangeability of the $\gamma_{j}$s still holds under model (6) or (7). This differs from the situation where prior knowledge exists to distinguish some baskets from others, thereby breaking the prior exchangeability assumption as discussed in Section 2.3.

The estimation of μ and $\sigma^{2}$ follows a similar logic as in the simple normal prior case. The number and weights of mixture components, *K* and w, may be prespecified or estimated from the data. Standard prior choices include a symmetric Dirichlet distribution prior for w conditional on *K*, and a zero-truncated Poisson distribution prior for *K* [38]. Since the dimensions of μ, $\sigma^{2}$, and w depend on *K*, posterior computation under this approach typically requires trans-dimensional Markov chain Monte Carlo [39]. To avoid such computational complexity, an alternative strategy is to fit multiple models with different values of *K* and select the most appropriate *K* based on a model selection criterion such as the deviance information criterion [15,40]. From a nonparametric Bayesian modeling perspective, one may set K=∞ to allow for flexibility. By further letting $w_{k}=v_{k}\prod_{l=1}^{k-1}\left(1-v_{l}\right)$ and $v_{l}∼\mathrm{Beta}\left(1,\zeta \right)$, G(θ) becomes a Dirichlet process mixture model [21,41].

#### 2.5.2. Bayesian Model Averaging

When it is reasonable to assume that the treatment has the same (transformed) response rate across multiple baskets, Bayesian model averaging can be utilized to facilitate information borrowing [16,22]. Let $M_{\ell }$ denote a partition of the *J* baskets into subsets. For example, when J=3 baskets, the possible partitions include $M_{1}=\left\{\left\{1,2,3\right\}\right\}$, $M_{2}=\left\{\left\{1,2\right\},\left\{3\right\}\right\}$, $M_{3}=\left\{\left\{1,3\right\},\left\{2\right\}\right\}$, $M_{4}=\left\{\left\{2,3\right\},\left\{1\right\}\right\}$, and $M_{5}=\left\{\left\{1\right\},\left\{2\right\},\left\{3\right\}\right\}$. Each partition constrains different subsets of the (transformed) basket-specific response rates to be equal. For example, under partition $M_{2}$, $\gamma_{1}$ must be equal to $\gamma_{2}$, but there is no constraint on $\gamma_{3}$. To perform Bayesian inference, one may specify a prior for the distinct values of the (transformed) response rates conditional on the partition, as well as an additional prior for the partition itself.

Conditional on a given partition, information is pooled among baskets belonging to the same subset, while no information is borrowed between baskets in different subsets. The (marginal) posterior distribution of $\gamma_{j}$ is a weighted average of its posteriors under different partitions,

$$\begin{array}{c} p\left(\gamma_{j}|\mathrm{data}\right)=\sum_{\ell }p\left(\gamma_{j}|M_{\ell },\mathrm{data}\right)\cdot P\left(M_{\ell }|\mathrm{data}\right), \end{array}$$

which represents a compromise between complete pooling and stratified analysis. The weights in this average correspond to the posterior probabilities of the partitions.

### 2.6. Operating Characteristics

The likelihood (1) and prior (3) on $\pi_{j}$ (or a transformation of $\pi_{j}$) allow one to compute the posterior distribution of $\pi_{j}$. In most cases, the posterior is not analytically available, and Monte Carlo methods are used to approximate the posterior [42,43]. For the hypothesis test in Equation (2), a commonly used criterion to reject the null hypothesis $H_{0j}$ is when the posterior probability $\mathrm{Pr}\left(\pi_{j}>\pi_{0j}|\mathrm{data}\right)>q_{j}$, where $q_{j}$ is a prespecified threshold that may differ across baskets.

It is common practice to evaluate the operating characteristics of a Bayesian procedure under the frequentist paradigm, as it provides insights into the procedure’s long-run average behavior in repeated practical use [44]. In the context of basket trials, such evaluations are useful for understanding the practical implications of different prior choices for $\pi_{j}$s. Often, a set of scenarios is considered in which the true response rates are specified for the baskets, hypothetical response data are generated under each scenario, and relevant operating characteristics are recorded over repeated simulations. Table 3 provides an illustration of some possible response rate scenarios with four baskets and a reference rate of 20% for every basket. The scenarios encompass different combinations of promising and nonpromising baskets. The treatment response rates may also vary across the promising (or nonpromising) baskets. In Table 3, Scenario 1 is a global null scenario in which the treatment is inefficacious in all baskets, Scenario 2 is a global alternative scenario in which the treatment is efficacious in all baskets, and Scenarios 3–6 are mixed scenarios in which the treatment is efficacious in some but not all baskets.

The type I error rate and power are the most pertinent operating characteristics for basket trials [45]. A type I error refers to the incorrect rejection of a true null hypothesis, which, for basket trials, means to select a nonpromising basket for further investigation in a large-scale phase III study. The *basket-specific type I error rate* refers to the probability of committing a type I error in a specific basket, whereas the *family wise type I error rate* (FWER) is the probability of committing a type I error in any of the baskets. Using computer simulations, these error rates can be approximated by the relative frequencies of making the corresponding errors in a large number of simulated trials. When the null hypothesis is false, the correct action is to reject the null and select a truly promising basket for further investigation. The *basket-specific power* refers to the probability of correctly selecting a promising basket. The *family wise power* (FWP) is defined in a few different ways. For example, the *disjunctive power* (FWP-D) is the probability of correctly selecting any promising baskets, while the *conjunctive power* (FWP-C) is the probability of correctly selecting all promising baskets [46]. For a quick summary of the statistical concepts pertaining to type I error rate and power, refer to Table 4.

In exploratory basket trials, strict type I error rate control is not enforced by the regulators and is often at the discretion of the sponsors. While a more lenient type I error rate is linked to increased power, it implies a higher chance of selecting a nonpromising basket for further development, increasing the cost associated with a drug development program that will ultimately fail. On the other hand, a more stringent type I error rate is associated with reduced power, which leads to an increased chance of missing a truly promising basket. Sponsors should carefully navigate the tradeoff between risk and benefit, determining appropriate decision criteria under limited sample size that align with their specific needs and objectives.

We illustrate the impact of information borrowing on the type I error rate and power through a simulation study based on the six scenarios in Table 3. Under each scenario, 1000 sets of hypothetical response data are generated with sample size of 20 patients for every basket. Suppose that the borrowing occurs at the logit response rate level with an adjustment for the reference rate, i.e., we let $\gamma_{j}=\mathrm{logit}\left(\pi_{j}\right)-\mathrm{logit}\left(\pi_{0j}\right)$. Three prior choices are considered for the $\gamma_{j}$s that lead to different degrees of borrowing:

$$\begin{array}{cccc} & (I):\; & & \gamma_{j}\overset{iid}{∼}N\left(0,100^{2}\right);\\ & (\mathrm{II}):\; & & \gamma_{j}|\mu ,\sigma^{2}\overset{iid}{∼}N\left(\mu ,\sigma^{2}\right),\;\mu ∼N\left(0,100^{2}\right),\;\sigma ∼\mathrm{Half}-N\left(3\right);\\ & (\mathrm{III}):\; & & \gamma_{j}|\mu ,\sigma^{2}\overset{iid}{∼}N\left(\mu ,\sigma^{2}\right),\;\mu ∼N\left(0,100^{2}\right),\;\sigma ∼\mathrm{Half}-N\left(0.3\right). \end{array}$$

Here, Half-N(A) represents a half-normal distribution with scale parameter *A*, which belongs to the half-*t* prior family discussed by Gelman [36]. Priors I, II and III correspond to no, moderate and strong borrowing, respectively.

Recall that the null hypothesis associated with basket *j*, $H_{0j}$, is rejected when $\mathrm{Pr}\left(\pi_{j}>\pi_{0j}|\mathrm{data}\right)>q_{j}$. These posterior probability thresholds are typically chosen to achieve certain desirable type I error rate. Since multiple hypotheses are tested simultaneously, it may be desirable to incorporate a notion of FWER control, which limits the chance of falsely selecting any nonpromising baskets for further investigation [46]. The first type of FWER control, called *weak control*, requires that the FWER is controlled when all of the *J* null hypotheses are simultaneously true. For the six scenarios considered in Table 3, weak control of the FWER requires that the FWER is controlled under Scenario 1, the global null scenario. Suppose for simplicity the same posterior probability threshold is used across all baskets. To achieve a FWER of 5% under Scenario 1, the threshold values are 0.982, 0.946 and 0.964 under Priors I, II and III, respectively. The second type of FWER control, called *strong control*, is more stringent. It requires the control of the FWER regardless of which and how many null hypotheses are true. For the scenarios considered, strong control of the FWER requires that the FWER is controlled under all six scenarios including the mixed scenarios. Note that this does not guarantee FWER control beyond these six scenarios, but we restrict our attention to the six scenarios for simplicity. To achieve a FWER of below 5% under all six scenarios, the required threshold values under Priors I, II and III are 0.982, 0.996 and 0.984, respectively.

Table 5 shows the simulation results with weak FWER control. From Table 5, information borrowing is beneficial when the treatment response rates are homogeneous across baskets. For example, in Scenario 2, borrowing leads to substantially increased basket-specific and family wise power. In this case, the stronger the borrowing, the larger the increase in power. When the response rates are heterogeneous, the performance of borrowing does not always compare favorably with that of no borrowing. For example, in Scenario 3, borrowing results in inflated type I error rate. In Scenario 6, strong borrowing results in lower power compared to no borrowing.

Table 6 reports the simulation results with strong FWER control. The issue of inflated type I error rate due to borrowing is mitigated by increasing the posterior probability thresholds. In the global alternative scenario, although less substantial, borrowing still leads to increased power. In the mixed scenarios, however, borrowing (especially strong borrowing) usually results in lower power compared to no borrowing.

In summary, in terms of operating characteristics, borrowing is beneficial when the response rates are homogeneous but may be unfavorable when the response rates are heterogeneous. For this reason, there has been some controversy about the usefulness of information borrowing in basket trials [47]. Our opinion is that borrowing is still useful. First, in the Bayesian framework, a prior serves as an expression of belief regarding which parameter values are deemed more plausible. When the prior is designed to encourage information borrowing, it implies a belief that the response rates are more likely to be homogeneous across baskets. Consequently, the performance in scenarios with homogeneous response rates should be given greater weight compared to that in scenarios with heterogeneous response rates. Second, Table 5 shows that under weak FWER control, moderate borrowing leads to considerable gain in power in the global alternative scenario without compromising much of the type I error rate and power in the mixed scenarios. In fact, with more sophisticated Bayesian modeling and judicious information borrowing, many recent methods achieve even more improvements in power while maintaining type I error rates at reasonable levels, even in the mixed scenarios [12,14,21]. To this end, we recommend setting up the statistical analysis to borrow information across baskets where the treatment is expected to exhibit similar behavior based on the drug mechanism. If there is uncertainty about the homogeneity of the true response rates, it is recommended to borrow information in a judicious manner.

### 2.7. Interim Analysis

Patient enrollment in clinical trials typically occurs sequentially. Therefore, when designing a clinical trial, it may be desirable to incorporate provisions for interim analyses of accumulating data, allowing for the possibility of early termination of the trial [48]. Oftentimes, basket trial designs include interim monitoring for futility [11,14,16]. At the *r*th interim analysis, if

$$\begin{array}{c} \mathrm{Pr}\left(\pi_{j}>{\tilde{\pi }}_{0j}|\mathrm{data}\;\mathrm{at}\;r\mathrm{th}\;\mathrm{interim}\right)<c_{jr}, \end{array}$$

patient accrual in basket *j* is halted, as the treatment is deemed inefficacious in this basket. Here, $c_{jr}$ is a prespecified threshold (e.g., 0.05), and ${\tilde{\pi }}_{0j}$ may be chosen based on both the reference rate $\pi_{0j}$ and a prespecified target response rate $\pi_{1j}$ (e.g., ${\tilde{\pi }}_{0j}=\left(\pi_{0j}+\pi_{1j}\right)/2$). Alternatively, the futility stopping rule can be based on the posterior predictive probability of success [48,49]. Early stopping rules have an impact on the operating characteristics of a design. For example, futility stopping rules reduce the expected number of patients enrolled and type I error rate, which can help avoid devoting too much resources to nonpromising baskets. However, they also result in a decrease in the power of finding the promising baskets.

Interim analyses can also be used to serve the purpose of adjusting the extent of information borrowing as the trial progresses. In Cunanan et al. [50], the authors proposed to assess the homogeneity of treatment effects across baskets in an interim analysis via Fisher’s exact test [51]. If homogeneity is not rejected, data across all baskets are pooled into one group in the final analysis, whereas otherwise each basket is analyzed individually. The critical value of the Fisher’s exact test statistic is a tuning parameter and is prespecified. As another example, Liu et al. [13] proposed to evaluate response rate heterogeneity in an interim analysis using Cochran’s Q test [52]. If homogeneity is not rejected, a Bayesian hierarchical mixture model is used to borrow information across baskets in the final analysis. Otherwise, each basket is investigated independently.

### 2.8. Non-Technical Summary

This section discusses several aspects of Bayesian methods for information borrowing in phase II basket trials. Key statistical considerations include setting up appropriate prior distributions for quantities that are deemed homogeneous across baskets, such as response rates of the investigational drug or their increments over the reference rates. We demonstrate the benefits of information borrowing through simple simulations and advocate for the use of Bayesian methods that lead to increased statistical power despite potential type I error rate inflation.

## 3. Basket in Phase I for Dose Optimization

Traditional cytotoxic oncology drugs (e.g., chemo-therapies) exert their efficacy through mechanisms that directly induce cell death, cancerous or not. Therefore, a higher dose leads to more cell death, which then leads to higher efficacy and toxicity. For this reason, the maximum tolerated dose (MTD) is considered optimal for patient care since it produces the highest efficacy among all the doses that can be tolerated. In a phase I oncology trial, simple statistical designs like 3+3 [53] and i3+3 [54] are routinely used to identify a single dose as the MTD at the end of the trial. However, this MTD-centric paradigam is now being challenged.

**Figure 3:**
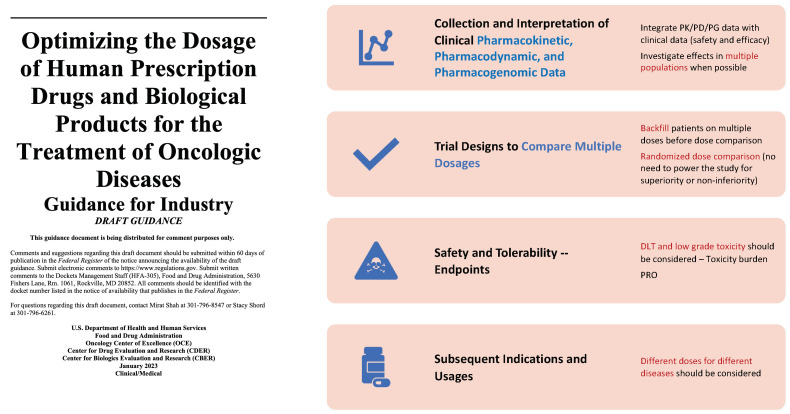
Summary of the FDA draft guidance on dose optimization.

Due to the explosive advancement in biological and genomics research since the human genome was sequenced in the early 2000s [55,56], oncology drugs have switched from directly eradicating cancer cells based on cytotoxic means to precisely targeting biological processes at the molecular level such as genetic and immune pathways. The vast success in PD-1 inhibitors [57] highlights the paradigm shift in oncology drug development. As a consequence, the design and conduct of phase I oncology trials are being transformed with the launch of the U.S. FDA’s Project Optimus [25], which aims to adapt the approach of clinical trials to the new realities of cancer treatment. Under this initiative, the FDA encourages the development and application of novel trial designs and statistical methods that attempt to identify the optimal dose of oncology drugs instead of the MTD. Several publications [58,59,60] and an FDA draft guidance [24] have called for changes to early phase clinical trial designs. See Figure 3 for a summary of the draft guidance.

**Figure 4:**
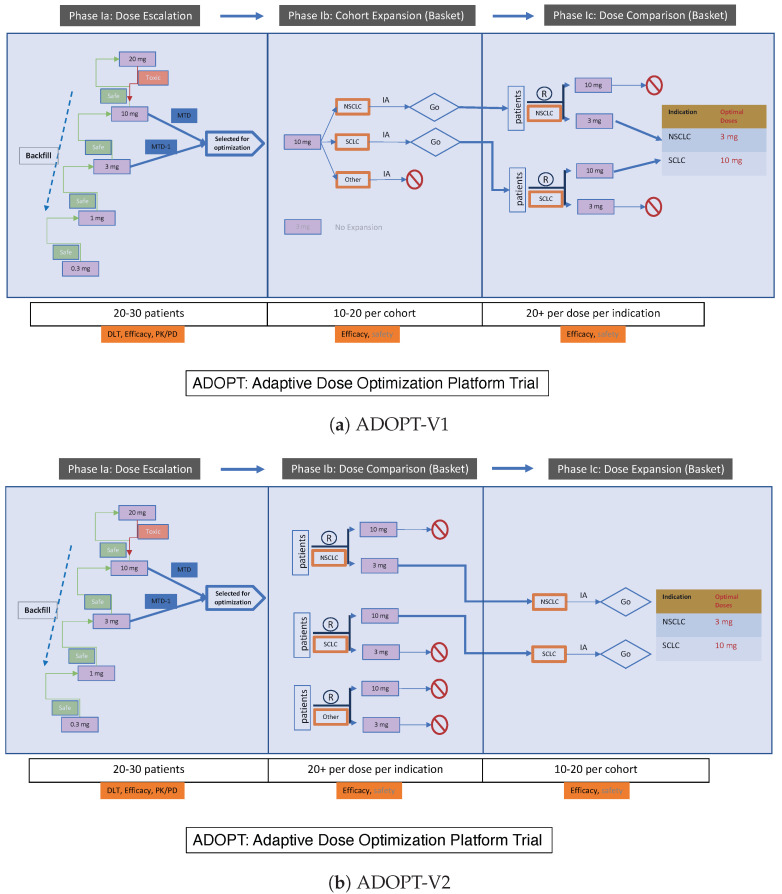
A stylized illustration of the Adaptive Dose Optimization Platform Trial (ADOPT). It consists of three seamless phases, Ia, Ib, and Ic. Phase Ia is for dose escalation. Phases Ib and Ic are basket trials for expansion and randomized dose comparison. IA stands for interim analysis. Novel features like backfill and integration of PK/PD data can be considered in phase Ia. The order of phases Ib and Ic may change depending on specific settings in practice, shown as the two versions V1 in (**a**) and V2 in (**b**). In the end, different indications may have different optimal doses. For example, 3mg for NSCLC and 10mg for SCLC are selected as the optimal doses.

Ji and Bi [61] proposed a new platform trial design for early phase dose optimization. The design, called ADOPT, standing for Adaptive Dose Optimization Platform Trial, is structured as a phase I trial consisting of three seamless sub-phases, Ia, Ib, and Ic. Two versions of ADOPT are presented in Figure 4, denoted as ADOPT-V1 and ADOPT-V2. In both versions, phase Ia represents an improved dose escalation study highlighted by novel features like patient backfill and the use of PK/PD data. At the end of phase Ia, doses 10 mg (the MTD) and 3 mg (the dose below MTD) are selected and sent to phases Ib and Ic for testing of efficacy. ADOPT-V1 (Figure 4a) applies the multi-arm two-stage (MATS) design [28] to phases Ib and Ic. Specifically, phase Ib expands the higher dose 10 mg in three indications, making it a basket-like study. At the end of phase Ib, an interim analysis is performed for each indication to determine whether the higher dose 10 mg shows promising efficacy in that indication. If yes, the indication is selected for a randomized comparison between 10mg and 3 mg in the subsequent phase Ic. Multiple indications may be selected for phase Ic, making it another basket-like study that also involves multiple doses. In other words, phases Ib/Ic constitute a double-basket trial. ADOPT-V2 (Figure 4b) reverses the order of dose expansion and randomized comparison. The two versions of ADOPT may be suitable for different drug development programs and mechanisms of action. For example, if it is strongly believed that the higher dose is more efficacious than the lower dose, ADOPT-V1 might be a better design since it only tests the lower dose (in phase Ic) when the higher dose demonstrates promising efficacy. Otherwise, ADOPT-V2 might be preferred, which allows randomized comparison between the two doses immediately after dose escalation in phase Ia.

The double-basket phases Ib and Ic in ADOPT offer opportunities for employing statistical methods that facilitate information borrowing across indications. Take ADOPT-V1 as an example, which utilizes the MATS design [28] for the double-basket phases. Let i=1 and 2 denote the higher and lower doses, 10 mg and 3 mg, respectively. Furthermore, let k=1 and 2 denote the two stages corresponding to phases Ib and Ic. Finally, let j=1,…,J denote the indications. In Figure 4a, J=3 corresponding to NSCLC, SCLC and other. The tuple (i,j,k) uniquely identifies an “arm” in the trial. For each arm (i,j,k), denote by $n_{ijk}$ the number of patients treated and $y_{ijk}$ the number of responders. Then, assume the following sampling model,

$$\begin{array}{cccc} \; & \mathrm{Stage}\;1: & \; & y_{1j1}|n_{1j1},\pi_{1j}∼\mathrm{Bin}\left(n_{1j1},\pi_{1j}\right),\\ \; & \mathrm{Stage}\;2: & \; & y_{ij2}|n_{ij2},\pi_{ij},D_{j1}=1∼\mathrm{Bin}\left(n_{ij2}\cdot D_{j1},\pi_{ij}\right), \end{array}$$

where $\pi_{ij}$ represents the true but unknown response rate of dose *i* in indication *j*, and $D_{j1}=1$ (or 0) represents that indication *j* is selected (or not selected) for further testing in stage 2. The goal of the double-basket phases is twofold: comparing $\pi_{ij}$ to an indication-specific reference rate $\pi_{0j}$ for both doses (proof of concept), and comparing $\pi_{1j}$ to $\pi_{2j}$ between the two dose levels (dose optimization). The MATS design utilizes the following Bayesian hierarchical model to borrow information across indications,

$$\begin{array}{cccc} \; & \mathrm{Transformation}: & \; & \gamma_{1j}=\mathrm{logit}\left(\pi_{1j}\right)-\mathrm{logit}\left(\pi_{0j}\right);\\ \; & \; & \; & \gamma_{2j}=\mathrm{logit}\left(\pi_{1j}\right)-\mathrm{logit}\left(\pi_{2j}\right);\\ \; & \mathrm{Prior}\;\mathrm{for}\;\gamma_{1j}: & \; & \gamma_{1j}|\mu_{1},\sigma_{1}^{2}\overset{iid}{∼}N\left(\mu_{1},\sigma_{1}^{2}\right);\\ \; & \mathrm{Prior}\;\mathrm{for}\;\gamma_{2j}: & \; & \gamma_{2j}|\mu_{2},\sigma_{2}^{2}\overset{iid}{∼}\mathrm{LogNormal}\left(\mu_{2},\sigma_{2}^{2}\right);\\ \; & \mathrm{Hyperpriors}: & \; & \mu_{1}∼\mathrm{Normal},\sigma_{1}^{2}∼\mathrm{Inv}-\mathrm{Gamma};\\ \; & \; & \; & \mu_{2}∼\mathrm{Normal},\sigma_{2}^{2}∼\mathrm{Inv}-\mathrm{Gamma}. \end{array}$$

Here, $\gamma_{1j}$ represents the response rate increment (on the logit scale) of the high dose over the reference rate in indication *j*. Modeling the $\gamma_{1j}$s as a random sample from a common normal distribution allows information borrowing of the response rate increments across indications. This is analogous to the idea illustrated in Equation (3) and Figure 1. Similarly, $\gamma_{2j}$ represents the response rate difference (on the logit scale) between the high and low doses in indication *j*. Again, the common log-normal prior for the $\gamma_{2j}$s facilitates information borrowing of the response rate differences. It is assumed that the response rate is increasing with the dose level, and thus the $\gamma_{2j}$s are restricted to be positive. However, when the assumption is unlikely to hold, a more neutral prior, such as a normal distribution allowing $\gamma_{2j}$s to be negative, may be considered.

### Non-Technical Summary

This section discusses the application of Bayesian methods for information borrowing in phase I dose optimization trials. Through reviewing the MATS design, we demonstrate how information borrowing can be incorporated in trials that involve multiple doses, indications, and stages.

## 4. Discussion and Future Directions

We have provided an overview of Bayesian methods for information borrowing in basket trials and have summarized the general components of these methods. For other aspects of basket trials, we refer interested readers to [62,63,64,65,66]. For example, Park et al. [62] performed a systematic literature search to identify clinical trials that had been proposed and conducted with a basket design. Kaizer et al. [63] offered more insights into the statistical considerations, in particular those related to the type I error rate. Pohl et al. [64] covered both Bayesian and frequentist methods with more emphasis on the variety of statistical models.

Statistical software that implements Bayesian methods for basket trials is scarce. Table 7 lists a few notable ones with the most comprehensive software being commercial.

To date, we have discussed the applications of basket designs in exploratory phase I and phase II trials. Confirmatory basket trials, on the other hand, require additional statistical considerations. For example, whether it is still appropriate to borrow information across baskets, and whether it is necessary to impose stringent control of the FWER [68,69]. Recent novel basket trial designs [70,71,72] shed some lights on the potential efficiency gain of confirmatory basket trials by adding a “pruning” step using external data and interim trial data to weed out unpromising indications and by performing a post-individual check after the final pooled analysis of data from all indications. In He et al. [72], the authors showed that such a design could improve the efficiency of the trial while still controlling the FWER. While the proposed design was not based on Bayesian models, the authors suggested that Bayesian techniques devised for exploratory basket trials may further improve the performance of their design.

None of the methods reviewed in this article consider borrowing on the basis of similarities between patients. In other words, if patient populations across two baskets are “similar”, it is more likely they will respond to the treatment similarly. The similarity of patients can be measured by the distance of their covariate distributions, which sets up a model framework for dependent distributions of covariates. This might be a future direction of statistical research for basket trials.

## 5. Conclusions

Basket trials allow simultaneous evaluation of an investigational drug in multiple patient subpopulations within a single study. Since patients across baskets receive the same treatment, it is sensible to borrow information across them to improve estimation of treatment effects in different baskets. Bayesian methods provide a natural choice to achieve information borrowing and are the focus of our review. An overarching theme across the reviewed methods is to assume the (transformed) response rates for different baskets arise from a common population distribution. This provides opportunities for Bayesian statisticians to set up priors for the transformed response rates that are essentially exchangeable and therefore enable information sharing in the estimation procedure. Simulation studies can be used to calibrate the decision criteria for efficacy evaluation to achieve desirable operating characteristics under information borrowing.

## Figures and Tables

**Table 1:** Data from the imatinib trial. Here, *y* represents the number of responders, and *n* is the total number of patients by sarcoma subtype.

Subtype	*y*	*n*	%
1. Angiosarcoma	2	15	13.3
2. Ewing	0	13	0.0
3. Fibrosarcoma	1	12	8.3
4. Leiomyosarcoma	6	28	21.4
5. Liposarcoma	7	29	24.1
6. MFH	3	29	10.3
7. Osteosarcoma	5	26	19.2
8. MPNST	1	5	20.0
9. Rhabdomyosarcoma	0	2	0.0
10. Synovial	3	20	15.0
Total	28	179	15.6

**Table 2:** Data from the vemurafenib trial. Here, *y* represents the number of responders, and *n* is the total number of patients by cancer cohort.

Cohort	*y*	*n*	%
1. ATC	2	7	28.6
2. ECD/LCH	6	14	42.9
3. CCA	1	8	12.5
4. CRC-V	1	26	3.8
5. CRC-VC	0	10	0.0
6. NSCLC	8	19	42.1
Total	18	84	21.4

**Table 3:** Examples of response rate scenarios used in simulations to evaluate methodologies for analyzing basket trials.

Scenario	Response Rates					Reference Rates			
	$\pi_{1}$	$\pi_{2}$	$\pi_{3}$	$\pi_{4}$		$\pi_{01}$	$\pi_{02}$	$\pi_{03}$	$\pi_{04}$
1 global null	20%	20%	20%	20%		20%	20%	20%	20%
2 global alternative	**35%**	**35%**	**35%**	**35%**		20%	20%	20%	20%
3 mixed	20%	**35%**	**35%**	**35%**		20%	20%	20%	20%
4 mixed	20%	20%	**35%**	**35%**		20%	20%	20%	20%
5 mixed	10%	20%	**30%**	**40%**		20%	20%	20%	20%
6 mixed	20%	20%	20%	**35%**		20%	20%	20%	20%

The values highlighted in bold represent the promising baskets.

**Table 4:** Summary of concepts pertaining to type I error rate and power in basket trials.

Concept	Description
**Type I error rate**	
Basket-specific	Probability that a specific nonpromising basket is falsely identified as promising
Family wise (FWER)	Probability that any nonpromising basket is falsely identified as promising
**Power**	
Basket-specific	Probability that a specific promising basket is correctly identified as promising
Family wise & disjunctive (FWP-D)	Probability that any promising basket is correctly identified as promising
Family wise & conjunctive (FWP-C)	Probability that all promising baskets are correctly identified as promising
**FWER control**	
Weak control	FWER is controlled when all baskets are nonpromising
Strong control	FWER is controlled regardless of which and how many baskets are nonpromising

**Table 5:** Operating characteristics under the six simulation scenarios with different degrees of borrowing. The posterior probability thresholds are calibrated to achieve weak control of the FWER under the global null scenario.

Scenario	Borrowing	% Reject				FWER	FWP-D	FWP-C
1		0.2	0.2	0.2	0.2			
	No	1.2	1.3	1.3	1.3	5.0	0.0	0.0
	Moderate	1.5	1.5	1.3	1.4	5.0	0.0	0.0
	Strong	2.2	2.3	2.3	2.2	5.0	0.0	0.0
2		**0.35**	**0.35**	**0.35**	**0.35**			
	No	25.6	25.4	25.6	26.0	0.0	69.6	0.2
	Moderate	54.0	52.9	54.7	55.5	0.0	87.0	18.9
	Strong	79.9	78.1	78.8	79.8	0.0	90.3	63.6
3		0.2	**0.35**	**0.35**	**0.35**			
	No	1.1	25.2	25.2	27.0	1.1	59.2	1.7
	Moderate	8.7	43.1	44.1	45.5	8.7	73.5	15.6
	Strong	33.9	61.6	62.7	62.1	33.9	74.9	47.7
4		0.2	0.2	**0.35**	**0.35**			
	No	1.2	1.6	25.3	26.4	2.8	45.6	6.1
	Moderate	5.5	4.9	34.6	37.0	8.8	54.1	17.5
	Strong	18.6	18.1	43.7	44.1	25.3	53.1	34.7
5		0.1	0.2	**0.3**	**0.4**			
	No	0.0	1.5	12.2	44.9	1.5	51.9	5.2
	Moderate	0.0	2.4	17.8	46.6	2.4	52.6	11.8
	Strong	1.9	8.7	21.5	38.4	8.8	41.7	18.2
6		0.2	0.2	0.2	**0.35**			
	No	1.0	1.5	1.2	27.0	3.7	27.0	27.0
	Moderate	3.2	2.8	2.4	27.0	7.1	27.0	27.0
	Strong	8.2	7.9	7.6	24.7	14.0	24.7	24.7

The values highlighted in bold represent the promising baskets.

**Table 6:** Operating characteristics under the six simulation scenarios with different degrees of borrowing. The posterior probability thresholds are calibrated to achieve strong control of the FWER under all six scenarios.

Scenario	Borrowing	% Reject				FWER	FWP-D	FWP-C
1		0.2	0.2	0.2	0.2			
	No	1.2	1.3	1.3	1.3	5.0	0.0	0.0
	Moderate	0.6	0.5	0.7	0.5	2.0	0.0	0.0
	Strong	0.1	0.1	0.1	0.1	0.3	0.0	0.0
2		**0.35**	**0.35**	**0.35**	**0.35**			
	No	25.6	25.4	25.6	26.0	0.0	69.6	0.2
	Moderate	40.0	39.4	40.4	42.1	0.0	75.8	9.3
	Strong	35.4	34.9	36.5	37.5	0.0	60.4	12.8
3		0.2	**0.35**	**0.35**	**0.35**			
	No	1.1	25.2	25.2	27.0	1.1	59.2	1.7
	Moderate	5.0	30.2	30.1	32.0	5.0	58.6	7.2
	Strong	5.0	21.3	20.5	21.7	5.0	37.5	7.4
4		0.2	0.2	**0.35**	**0.35**			
	No	1.2	1.6	25.3	26.4	2.8	45.6	6.1
	Moderate	2.7	2.0	23.3	23.4	4.5	38.6	8.1
	Strong	2.2	1.3	10.1	10.6	3.0	16.0	4.7
5		0.1	0.2	**0.3**	**0.4**			
	No	0.0	1.5	12.2	44.9	1.5	51.9	5.2
	Moderate	0.0	1.5	10.6	33.3	1.5	39.3	4.6
	Strong	0.0	0.4	3.0	9.9	0.4	11.5	1.4
6		0.2	0.2	0.2	**0.35**			
	No	1.0	1.5	1.2	27.0	3.7	27.0	27.0
	Moderate	1.4	1.1	1.1	16.9	3.3	16.9	16.9
	Strong	0.5	0.4	0.4	4.0	1.2	4.0	4.0

The values highlighted in bold represent the promising baskets.

**Table 7:** Selected software packages that implement Bayesian methods for basket trials.

Software Name	Type	Description
R Package: basket (Ver. 0.10.11)	Open source	Implements the multi-source exchangeability model in Hobbs and Landin [16] and Kaizer et al. [67]
R Package: bmabasket (Ver. 0.1.2)	Open source	Implements the Bayesian model averaging approach in Psioda et al. [22]
R Package: bhmbasket (Ver. 0.9.5)	Open source	Implements the Bayesian hierarchical modeling approaches in Berry et al. [11] and Neuenschwander et al. [12]
Website: https://trialdesign.org/ (accessed on 22 December 2023)	Free	Implements the calibrated Bayesian hierarchical model in Chu and Yuan [14]
East Bayes (https://www.cytel.com/software/east-bayes/ (accessed on 22 December 2023))	Commercial software carried by Cytel, Inc. (Cambridge, MA, USA)	Compares up to four different basket trial designs
